# Translocation of Ultrafine Particles

**DOI:** 10.1289/ehp.114-a211b

**Published:** 2006-04

**Authors:** Abderrahim Nemmar, Peter H.M. Hoet, Benoit Nemery

**Affiliations:** Laboratory of Pneumology, Unit of Lung Toxicology, Katholieke Universiteit Leuven, Leuven, Belgium, E-mail: abderrahim.nemmar@med.kuleuven.ac.be

We read with great interest the article by Geiser et al. (2005) on the mechanism of translocation of ultrafine particles (UFPs) across cellular membranes *in vivo* in rats following inhalation and *in vitro* using porcine pulmonary macrophages and human red blood cells.

We are delighted to see this study that vindicates our hypothesis that translocation of UFPs is a possible pathway for the cardiovascular effects of particulate air pollution. A few years ago we reported extrapulmonary translocation of UFPs after intratracheal instillation in hamsters (Nemmar et al. 2001) and after inhalation in healthy human volunteers (Nemmar et al. 2002a), suggesting an alternative and/or a complementary explanation for the extrapulmonary effects of particles.

In their study, Geiser et al. (2005) very elegantly provided novel morphologic data showing the occurrence of translocation of UFPs, and they also reported—for the first time—that this translocation did not occur by endocytic processes but rather by diffusion or adhesive interactions. However, we noted with some surprise that the authors cited Brown et al. (2002) when referring to previous studies on the occurrence of UFP translocation. Indeed, Brown et al. (2002) studied the deposition and clearance of an ultrafine (60 nm) technetium-99m–labeled aerosol in human volunteers after 2 hr, and found no significant radioactivity in the liver (1.3 ± 1.2%). This activity was attributed to scatter from the lung and/or overlap of lung parenchyma in the liver. Consequently, Brown et al. (2002) excluded the occurrence of translocation and, although they did not measure radioactivity in blood, they challenged our conclusion that UFPs (5–10 nm) could pass from the lungs into blood and extrapulmonary organs (Nemmar et al. 2002a). Therefore, for the sake of accuracy, Geiser et al. (2005) should have referred to our study (Nemmar et al. 2002a) rather than that of Brown et al. (2002).

Geiser et al. (2005) provided micrographs of fluorescent polystyrene particles taken up by macrophages (Figure 3) or red blood cells (Figure 4). It is not clear whether these polystyrene particles contained surface charges, for example, carboxylate-modified (negatively charged) or amine-modified (positively charged), although Geiser et al. (2005) briefly discussed the possible effect of surface charge. This aspect is important; we (Nemmar et al. 2002b) and others (Silva et al. 2005) have reported that hemostasis may be affected by the intravenous or intratracheal administration of UFPs and also established that this phenomenon is dependent on the surface properties of the particles. Thus, only positively charged amine-modified particles led to a marked increase in prothrombotic tendency, which we showed to result, at least in part, from platelet activation

In conclusion, although the article by Geiser et al. (2005) adds a significant amount of information to the literature related to the extrapulmonary effect of inhaled particles, this issue needs to be clarified in more detail. Therefore, we look forward to this group and others providing more detailed quantification of the proportion of inhaled UFPs that can be found in extrapulmonary organs.
